# Supplementary material for: Studies on Annihilation and Coreactant Electrochemiluminescence of Thermally Activated Delayed Fluorescent Molecules in Organic Medium
Source: Molecules. 2022 Nov 2;27(21):7457. doi: 10.3390/molecules27217457 (PMC9658960; doi:10.3390/molecules27217457)
Supplement: Supplementary file 1 [file molecules-27-07457-s001.zip › molecules-2000084-supplementary.pdf]

Supporting information

Studies on Annihilation and Coreactant Electrochemiluminescence of Thermally Activated Delayed Fluorescent Molecules in Organic Medium

Ping Huang †, Xingzi Zou †, Zhiyun Xu, Yanting Lan, Lijuan Chen *, Baohua Zhang * and Li Niu

Center for Advanced Analytical Science, Guangzhou Key Laboratory of Sensing Materials & Devices, School of Chemistry and Chemical Engineering, Guangzhou University, Guangzhou 510006, China

* Correspondence: gdchenlj@gzhu.edu.cn (L.C.); ccbhzhang@gzhu.edu.cn (B.Z.)

† These authors contributed equally to this work.

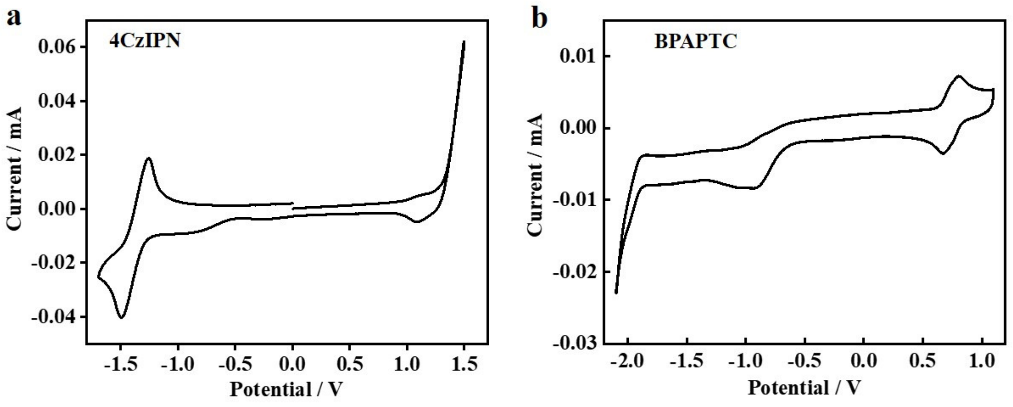

Figure S1. The CV curves of 0.1 mM 4CzIPN DCM solution (a) and 0.1 mM BPAPTC DCM solution (b) (vs. Ag/Ag+).

Notation: The electroredution current starting at ca. -0.75 V for BPAPTC should not be ascribed to electroreduction of BPAPTC itself. Most likely, it is due to the electrochemical reduction of trace water or others dissoved in the DCM solvent.

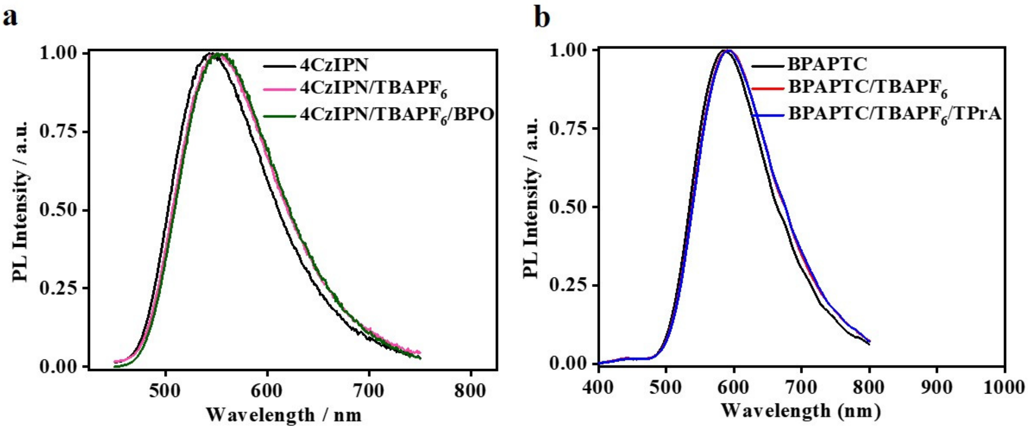

Figure S2. (a) Normalized PL spectra of 0.1 mM 4CzIPN in DCM, 0.1 mM 4CzIPN in DCM with 0.1 M $TBAPF_6$, 0.1 mM 4CzIPN in DCM with 0.1 M $TBAPF_6$ and 25 mM BPO. (b) Normalized PL spectra of 0.1 mM BPAPTC in DCM, 0.1 mM BPAPTC in DCM with 0.1 M $TBAPF_6$, 0.1 mM BPAPTC in DCM with 0.1 M $TBAPF_6$ and 25 mM TPrA.
